# Reference Values for Respiratory Impedance in Bulgarian Children Aged 2–8 Years Using the Forced Oscillation Technique (FOT)

**DOI:** 10.3390/children12070957

**Published:** 2025-07-21

**Authors:** Plamena Stoimenova, Stoilka Mandadzhieva, Blagoi Marinov

**Affiliations:** Department of Pathophysiology, Medical University of Plovdiv, 4002 Plovdiv, Bulgaria; smandadzhieva@pathophysiology.info (S.M.); bmarinov@pathophysiology.info (B.M.)

**Keywords:** forced oscillation technique, determinants, reference values, children, pulmonary function testing

## Abstract

**Background/Objectives**: The forced oscillation technique (FOT) is a non-invasive, effort-independent method for assessing respiratory mechanics and is particularly suited for young children who cannot reliably perform spirometry. This study aimed to evaluate the main anthropometric determinants of respiratory impedance parameters—resistance (Rrs) and reactance (Xrs)—in healthy Bulgarian children aged 2 to 8 years. **Methods**: A total of 100 healthy children were evaluated using a commercially available device at oscillation frequencies of 5, 11, and 19 Hz. Anthropometric data were collected, and FOT measurements were conducted following ATS/ERS guidelines. Stepwise multiple linear regression was applied to identify predictors of Rrs and Xrs. **Results**: Height (mean height of the children: 113.89 ± 8.46 cm) emerged as the most significant determinant of both Rrs and Xrs across all frequencies with a moderate inverse correlation observed between Rrs at 5 Hz and height (r = −0.446; *p* < 0.001). Weight additionally influenced reactance at 5 Hz. The mean R5–19 was 0.55, but no significant associations with height or weight were found. Stepwise regression confirmed height as the sole consistent predictor, while sex and age had no significant effect. **Conclusions**: This study is the first to present the determinants of key FOT parameters in a population of Bulgarian children. Height was identified as the strongest predictor of respiratory impedance and should be prioritized in the development of reference values for children under 8 years old. These findings reinforce the clinical utility of FOT in early childhood.

## 1. Introduction

The forced oscillation technique (FOT) is a non-invasive, effort-independent method for assessing respiratory mechanics and is particularly valuable in subjects who are unable to perform traditional pulmonary function tests like spirometry [1]. Its simplicity and minimal cooperation requirements make it especially suitable in early childhood, where standard tests are often not feasible due to a lack of coordination or understanding. By superimposing small pressure oscillations over normal tidal breathing, FOT provides detailed insights into lung function without requiring forced expiratory maneuvers [2].

The technique is increasingly recognized for its value in the diagnosis and monitoring of various pulmonary conditions, including asthma, bronchiolitis, cystic fibrosis, and interstitial lung diseases. It has been found especially effective in identifying early airway injury (before clinically relevant symptoms) and in tracking disease progression or treatment responses over time [3,4,5,6,7].

FOT assesses respiratory mechanics by applying small pressure oscillations—usually through a mouthpiece—at varying frequencies to the respiratory system during tidal breathing. These oscillations allow the calculation of respiratory impedance, which consists of two main components: resistance (Rrs) and reactance (Xrs). Resistance reflects the opposition to airflow in the airways [8,9], while reactance represents the elastic and inertial properties of the respiratory system [10,11]. Both parameters are sensitive to changes in airway caliber, lung compliance, and breathing frequency, making them valuable indicators of respiratory health [12].

However, the accurate interpretation of FOT results requires comparison with reference values, which must be appropriate for the specific population and equipment used. This highlights the essential need for reference equations that take into account the main determinants such as age, height, gender, and population-specific characteristics. While several datasets for children have been published, they mainly use impulse oscillometry (IOS) [13,14,15,16].

In recent years, multiple studies have focused on establishing reference values for children using pseudorandom noise as the forcing signal. Among the most widely used reference equations for respiratory impedance parameters are those developed by Calogero et al., based on a population of healthy Italian children. The results show a high degree of consistency with the equations published by Hall et al. in an Australian pediatric cohort. Both studies used measurements at 6, 8, and 10 Hz, corresponding to the frequencies applied in impulse oscillometry [17,18,19].

In 2022, Ducharme et al. introduced updated reference equations for Canadian children aged 3 to 17 years, based on oscillometry measurements performed at 5, 11, and 19 Hz, reflecting the frequency settings of their equipment. These equations expand the applicability across age ranges and facilitate comparisons across different technical protocols [20].

These types of studies are crucial in enabling the accurate interpretation of FOT results and in distinguishing between normal and pathological findings in clinical settings.

Although FOT is recognized as a valuable tool in assessing respiratory function, its clinical application in Bulgaria remains relatively limited. Expanding access and standardizing protocols across centers would enhance diagnostic capabilities, especially in young children who are underserved by traditional testing methods.

While the relationship between anthropometric measures—particularly height—and FOT parameters is well documented, there is currently a lack of population-specific data for Bulgarian children. This study addresses that gap by presenting the first set of normative values for respiratory impedance in Bulgarian children aged 2 to 8 years. These findings aim to lay the groundwork for incorporating FOT more widely into pediatric respiratory evaluation in both clinical and research settings.

## 2. Materials and Methods

### 2.1. Participants

In this study, we recruited a cohort of 100 healthy preschool and young children who had no history of bronchial asthma nor any previously reported episodes of wheezing or other chronic respiratory conditions (Table 1). To ensure the accuracy and reliability of the respiratory function measurements, we excluded children who had experienced any signs of respiratory tract infections or symptoms, such as coughing and nasal congestion, at least two weeks preceding the assessment.

All participants included in the final analysis were Caucasian and were enrolled from kindergartens and primary schools located in the Pazardzhik region of southern Bulgaria. The recruitment of children from the general population, rather than from clinical settings, was meant to establish a statistically representative cohort of healthy children within this demographic group.

Prior to inclusion in the study, the purpose and procedures were explained in detail to the parents and legal guardians of each child. Efforts were made to ensure a child-friendly, stress-free testing environment during all measurements, particularly given the young age of the study population. Participation was strictly voluntary, and written informed consent was obtained for every child.

### 2.2. FOT Methodology

All participants were assessed on site, in kindergartens and primary schools located in Pazardzhik. The main parameters, Rrs and Xrs, were measured according to American Thoracic Society/European Respiratory Society (ATS/ERS) recommendations [21], using a commercially available device implementing the FOT (Resmon PRO FULL (V3), RESTECH Srl, Milano, Italy), which generates an oscillatory sound signal at multiple frequencies: 5, 11, and 19 Hz.

A daily calibration was performed as recommended prior to each testing session using a manufacturer-supplied calibration device to ensure the accuracy and consistency of the measurements.

**Figure 1:**
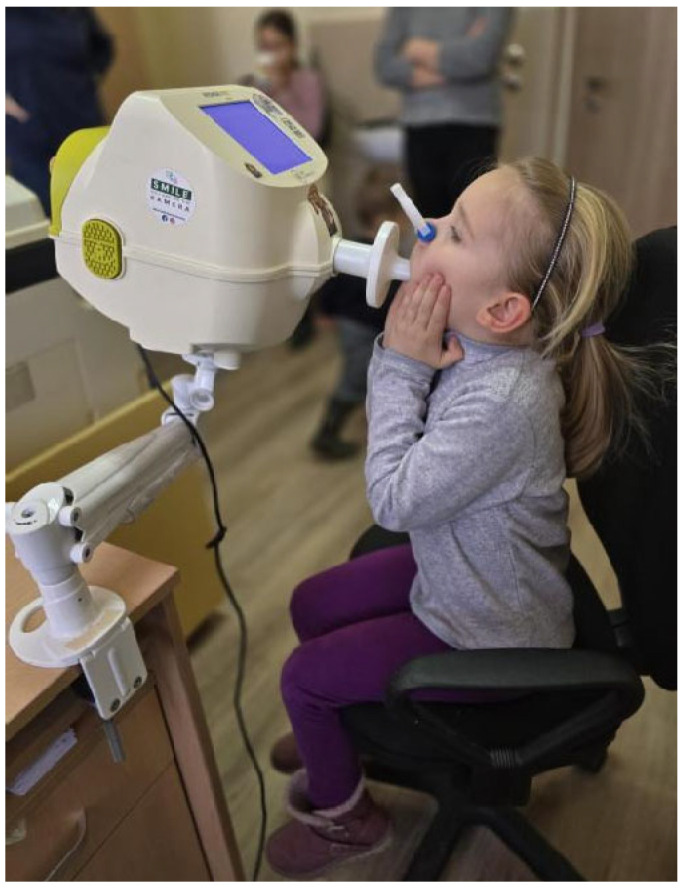
A 5-year-old girl correctly performing the forced oscillation technique (original data).

Each child performed the test in a seated position, with a straight back and their head held in a neutral or slightly extended position. To ensure proper airflow and minimize air leakage, a nose clip was placed on each child, and they were instructed to breathe quietly through a mouthpiece fitted with an antibacterial filter. To reduce upper airway shunting and improve measurement reliability, the cheeks and mouth floor were supported either by the examiner or, in the case of younger children, by a parent (Figure 1) [1,22,23].

For each child, at least three acceptable measurements were performed. Each measurement included a minimum of six valid tidal breaths, following the manufacturer’s recommendations. Quality control was applied to all recordings. Data was reviewed and only recordings without artifacts—such as coughing, talking, or crying—were included. All accepted data met predefined technical criteria to ensure consistency and reliability.

From each recording, values for respiratory resistance (Rrs) and respiratory reactance (Xrs) were extracted at each of the three tested frequencies. These parameters served as the primary indicators for evaluating respiratory mechanics within the studied population.

The Resmon PRO FULL (V3) device used in this study is a highly advanced and increasingly adopted device designed for accurate oscillometric measurements across multiple frequencies. Its child-friendly interface, real-time feedback, and built-in quality control features make it particularly well suited for use in preschool and early school-aged children, supporting its expanding role in both clinical practice and research involving pediatric respiratory assessment.

### 2.3. Statistical Methods

The collected data were statistically analyzed using SPSS software, version 20.0. The analysis included descriptive statistics, as well as variance, correlation, regression, and comparative analyses, to explore relationships between the forced oscillation technique (FOT) parameters and anthropometric variables. Stepwise multiple linear regression models were applied to identify the most significant predictors/determinants of respiratory resistance and reactance, with height, weight, gender, and age entered as independent variables. These models helped determine the degree to which each factor contributed to the variation in respiratory mechanics across the pediatric population. A *p*-value of less than 0.05 was considered statistically significant.

The study protocol was reviewed and approved by the Ethics Committee at the Medical University of Plovdiv, Bulgaria. The study was conducted in accordance with the principles outlined in the Declaration of Helsinki and all applicable national regulations concerning research involving human subjects.

## 3. Results

**Figure 2:**
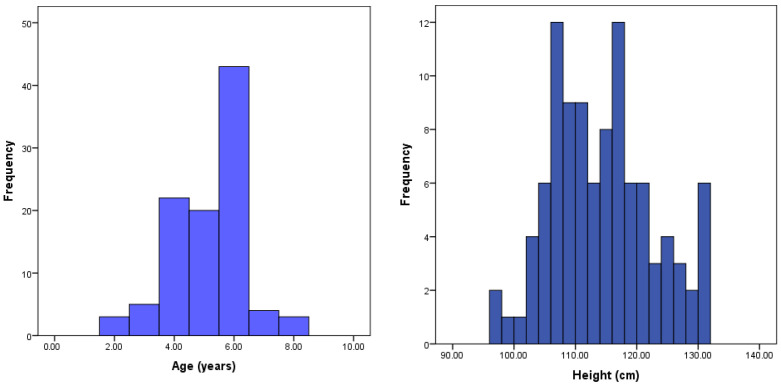
Distribution of the children involved in the research according to age and height.

A total of 100 children, ranging in age from 2 to 8 years, were included in the study sample. There was a slight predominance of boys, who made up 56% of the sample, compared to 44% girls. The distributions of age and height within the cohort are illustrated in Figure 2, providing a clear overview of the developmental spread across the sample, and the demographic and anthropometric characteristics of the study participants are illustrated in Table 1.

A significant positive correlation was found between age and height (r = 0.591) and between age and weight (r = 0.410), reflecting expected growth patterns in early childhood. The strongest association was between height and weight (r = 0.790), consistent with the typical parallel increase in stature and body mass.

Correlation coefficients between resistance and reactance and the anthropometric variables—age, height and weight—are presented on Table 2. The parameters are analyzed separately for boys and girls. Thе breakdown shows clearly how the FOT measures relate to growth parameters across sexes.

**Figure 3:**
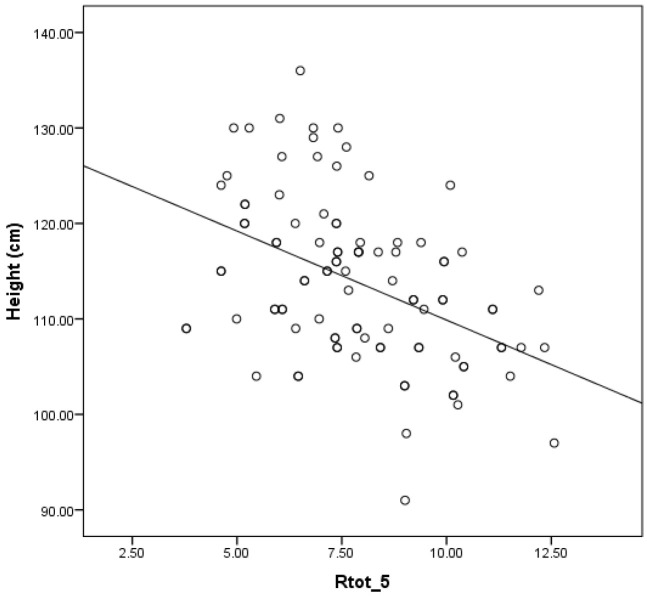
Relationship between height and resistance (Rrs).

A moderate inverse correlation exists between the mean whole-breath resistance at 5 Hz (Rtot 5) and height in children (r = −0.446; *p* < 0.001), as seen in Figure 3.

**Figure 4:**
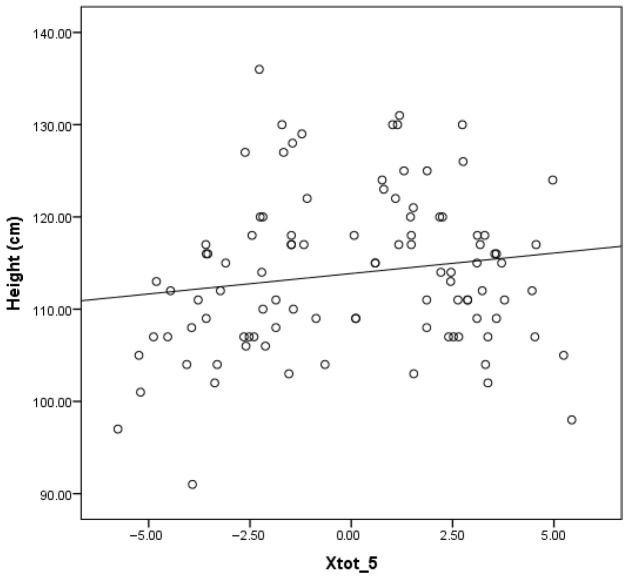
Relationship between height and reactance (Xrs).

In contrast to the findings related to the mean whole-breath resistance at 5 Hz (Rtot 5), the correlation presented in Figure 4 demonstrates a weak but statistically significant positive relationship between the mean whole-breath reactance at 5 Hz (Xtot 5) and height (r = 0.153; *p* = 0.014). This suggests that, with increasing height, there is a slight upward trend in Xtot 5 values.

There was no significant association between Rtot 5 or Xtot 5 and sex in our population of children aged 2 to 8 years, indicating that these respiratory impedance parameters are not influenced by gender within this age group.

**Figure 5:**
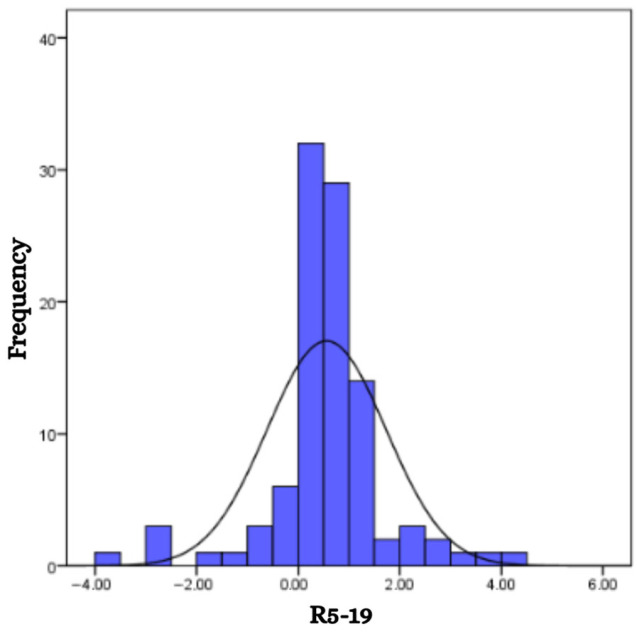
Frequency distribution of patients according to R5-19 results.

The mean value of the difference in respiratory resistance between 5 Hz and 19 Hz (R5–19) for the studied group was 0.55, with a range from −3.82 to 4.46 (Figure 5). No significant association was found between R5–19 and either the sex or the age of the children, although slight variations in mean values were observed between boys (0.57) and girls (0.53).

**Figure 6:**
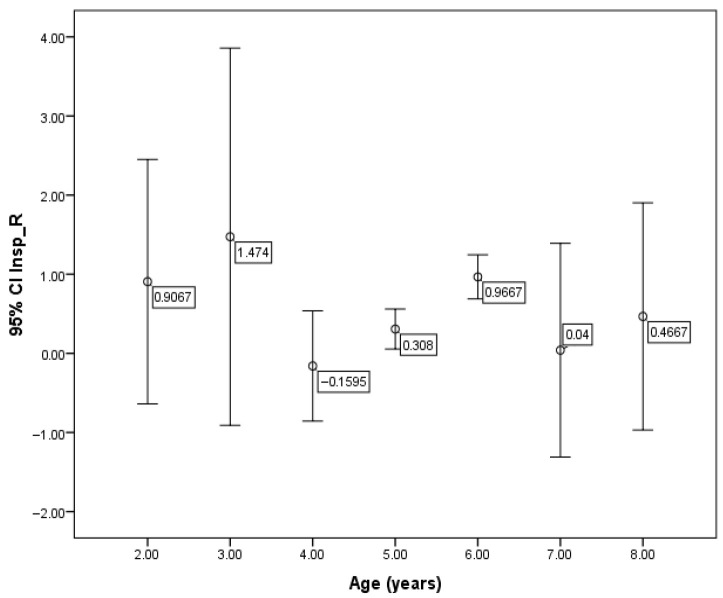
Mean values of R5-19 according to age.

Although no overall correlation between R5-19 and age was found, a significant difference in R5–19 values was observed across different age groups (*p* = 0.003) (Figure 6). However, no significant associations were detected between R5-19 and either weight or height in the children studied.

A multiple stepwise regression analysis was performed using the lowest recorded value for each child, considering both natural and logarithmic transformations of the variables: height, weight, age, and sex. The analysis revealed that height was the only significant predictor across all measured parameters, with one exception—reactance at an oscillating frequency of 5 Hz—where both height and weight emerged as significant predictors. For all other variables, neither sex nor age contributed meaningfully to the predictive models. These findings suggest that height plays a central role in influencing the physiological outcomes examined, whereas age and sex appear to have minimal predictive value in this context. A summary of the regression results is provided in Table 3.

## 4. Discussion

This study represents the first comprehensive effort to identify the key determinants of respiratory impedance parameters—resistance (Rrs) and reactance (Xrs)—obtained via the forced oscillation technique (FOT) in the pediatric population of Bulgaria. Height was identified as the primary determinant of both respiratory resistance and reactance across all tested frequencies and as a superior predictor of respiratory impedance compared to other commonly used parameters such as age, weight, or sex [17,19,24,25]. Although these additional factors are often included in lung function models, their incorporation did not significantly enhance the predictive accuracy of our regression analyses.

These findings are in line with well-documented physiological patterns, where somatic growth, particularly increases in height, corresponds with the expansion of airway caliber and lung volumes during childhood. As the lungs and airways grow, airway resistance tends to decrease, while elastic recoil and overall compliance (reflected in reactance) change accordingly. These developmental trends are especially important in early life, when lung growth is rapid and non-linear [12].

In the absence of region-specific normative data, this study fills in a critical gap by providing population-relevant reference values for respiratory impedance measured via FOT. The results offer a valuable foundation for clinical interpretation and future research in pediatric respiratory assessment.

The inverse relationship observed between Rtot 5 and height is physiologically consistent with normal lung growth and airway development during early childhood [26,27]. This developmental trajectory is well documented and aligns with established pediatric respiratory physiology. The trend of decreasing Rtot 5 as height increases supports the reliability of this parameter as a sensitive and developmentally appropriate marker for assessing airway function in young children [12].

Moreover, these findings highlight the clinical utility of FOT in pediatric populations, particularly in younger children who may not reliably perform conventional spirometry due to age or cognitive limitations [1,28]. This technique provides a non-invasive, effort-independent assessment of respiratory mechanics, allowing for the objective monitoring of airway resistance even in minimally cooperative patients. These attributes make FOT especially valuable for the early detection and longitudinal tracking of respiratory conditions such as asthma, bronchopulmonary dysplasia, or post-infectious airway obstruction in preschool-aged children [8,9,29].

Indeed, height was the only statistically significant predictor for Rrs across all frequencies tested. This finding is consistent with many other articles that also reported inverse correlations between height and resistance in healthy children [30,31,32,33,34,35,36]. Although our regression intercepts differ slightly from those reported by Klug and Bisgaard [37], this discrepancy primarily reflects differences in baseline values (offsets), likely due to variations in measurement techniques, equipment calibration, or sample characteristics. Importantly, the slope of the resistance–height relationship in our study closely aligns with their results, supporting the universality of the developmental trend across different cohorts.

In contrast to the inverse relationship observed for Rtot 5, a weak but statistically significant positive correlation was found between Xtot 5 and height. This suggests that as children grow taller, Xtot 5 values become less negative, reflecting improved compliance and reduced peripheral airway resistance—expected features in normal lung development. This trend is supported by prior research showing that reactance increases (becomes less negative) with lung growth in children, which identifies height as key determinant of reactance values in pediatric populations [13,38].

Our findings align with previous studies reporting no significant sex-related differences in respiratory impedance parameters measured via FOT in young children. For instance, Dencker et al. [13] found that gender did not significantly influence resistance or reactance values in children under 10 years of age. This consistency is likely due to the minimal physiological differences in lung size, thoracic structure, and airway mechanics between boys and girls prior to puberty.

In our cohort of children aged 2 to 8 years, the absence of sex-specific trends in Rrs and Xrs supports the idea that sexual dimorphism in lung function typically becomes evident only during and after puberty, driven by divergent growth patterns and hormonal changes [13]. Therefore, the lack of significant sex-related variation in this age group justifies the application of shared reference equations for both sexes in clinical and research settings.

The frequency-dependent change in resistance (R5–19), reflecting small airway resistance, showed no significant correlation with sex, age, height, or weight in our cohort, consistent with findings from De et al. [39]. In contrast, however, De et al. reported a clear decrease in R5–19 values with increasing age, suggesting a more pronounced age-related decline in small airway resistance. This difference may reflect variations in study populations, measurement techniques, or age distribution. Overall, these findings underscore the complexity of interpreting R5–19 in early childhood and the potential influence of subtle developmental factors.

The present analysis contributes to the growing body of research on FOT implementation in pediatric populations and adds important data from Eastern Europe, where such studies remain limited. By addressing the current lack of region-specific reference values, this work supports efforts to expand access to early diagnostic tools for young children.

Moreover, conducting the assessments in a non-clinical, community-based setting demonstrates the feasibility of applying FOT for broader screening and monitoring purposes. Its practicality and reliability strengthen the case for integrating FOT into routine pediatric respiratory evaluation, particularly in settings where traditional spirometry is less feasible.

## 5. Conclusions

This study confirms that height is the most significant anthropometric determinant of respiratory resistance, aligning with established physiological understanding and previous research. While sex and age showed no consistent linear relationship with Rrs and Xrs, significant group differences suggest developmental influences, particularly in early childhood. The development of population-specific reference equations is essential in the accurate interpretation of FOT results in both clinical and research settings.

## Figures and Tables

**Table 1:** Demographic and anthropometric characteristics of the study participants.

Variable		Value
Gender	Boys	56 (56.0%)
	Girls	44 (44.0%)
Weight (kg)	Mean ± SD (range)	20.4 ± 4.6 (12–37)
Height (cm)	Mean ± SD (range)	113.89 ± 8.46 (91–136)
BMI	Mean ± SD (range)	15.55 ± 2.11 (11.60–28.20)

**Table 2:** Correlation matrix for resistance and reactance and the anthropometric variables by gender.

	Boys			Girls			Combined (Boys and Girls)		
	Age	Height	Weight	Age	Height	Weight	Age	Height	Weight
R5 Hz	−0.227 *p = 0.092*	−0.448 *p = 0.001*	−0.121 *p = 0.373*	−0.423 *p = 0.004*	−0.452 *p = 0.002*	−0.358 *p = 0.017*	−0.308 *p = 0.002*	−0.446 ***p < 0.001***	−0.221 *p = 0.027*
R11 Hz	−0.205 *p = 0.129*	−0.451 ***p < 0.001***	−0.217 *p = 0.108*	−0.466 *p = 0.001*	−0.442 *p = 0.003*	−0.304 *p = 0.045*	−0.318 *p = 0.001*	−0.452 ***p < 0.001***	−0.251 *p = 0.012*
R19 Hz	−0.203 *p = 0.134*	−0.427 *p = 0.001*	−0.272 *p = 0.042*	−0.476 *p = 0.001*	−0.459 *p = 0.002*	−0.281 *p = 0.065*	−0.318 *p = 0.001*	−0.443 ***p < 0.001***	−0.270 *p* = 0.007
Х5 Hz	0.591 ***p < 0.001***	0.117 *p = 0.392*	0.056 *p = 0.683*	0.473 ***p < 0.001***	0.180 *p = 0.242*	0.111 *p = 0.471*	0.555 ***p < 0.001***	0.153 *p = 0.128*	0.084 *p = 0.405*
Х11 Hz	0.634 ***p < 0.001***	0.093 *p = 0.497*	−0.025 *p = 0.856*	0.430 *p = 0.002*	0.207 *p = 0.178*	0.147 *p = 0.340*	0.557 ***p < 0.001***	0.142 *p = 0.160*	0.053 *p = 0.602*
Х19 Hz	0.580 ***p < 0.001***	0.223 *p = 0.099*	0.139 *p = 0.307*	0.523 ***p < 0.001***	0.364 *p = 0.015*	0.290 *p = 0.057*	0.555 ***p < 0.001***	0.269 *p = 0.007*	0.195 *p = 0.052*

**Table 3:** Multiple stepwise regression to identify predictors for reactance and resonant frequency variables.

Model		Unstandardized Coefficients		Standardized Coefficients	T	*p*
		B	SE	Beta		
R5 Hz	Height	−0.173	0.034	−0.722	−5.021	0.000
	Weight	0.154	0.064	0.349	2.427	0.017
R11 Hz	Height	−0.107	0.021	−0.452	−5.010	0.000
R19 Hz	Height	−0.084	0.017	−0.443	−4.894	0.000
Х5 Hz	Height	−0.087	0.036	−0.252	−2.441	0.016
Х11 Hz	Height	−0.054	0.020	−0.273	−2.675	0.009
Х19 Hz	Height	0.441	0.069	0.543	6.394	0.000

Note: B, unstandardized regression coefficient; SE, standard error of the coefficient; Beta, standardized regression coefficient; T, T-statistic; *p*, *p*-value (significance level).

## Data Availability

The datasets presented in this article are not readily available due to ethical personal data sharing restrictions. Requests to access the datasets should be directed to the corresponding author.

## Abbreviations

The following abbreviations are used in this manuscript:

FOT	Forced oscillation technique
Rrs	Respiratory resistance
Xrs	Respiratory reactance
R5–19	Difference in respiratory resistance between 5 Hz and 19 Hz
Rtot 5	Mean resistance of the whole breath at 5 Hz
Xtot 5	Mean reactance of the whole breath at 5 Hz
